# Phosphorothioate DNA Stabilized Fluorescent Gold and Silver Nanoclusters

**DOI:** 10.3390/nano5020804

**Published:** 2015-05-19

**Authors:** Daniel S. Weadick, Juewen Liu

**Affiliations:** Department of Chemistry, Waterloo Institute for Nanotechnology, Waterloo, ON N2L 3G1, Canada; E-Mail: danweadick@gmail.com

**Keywords:** DNA, phosphorothioate, luminescence, nanoclusters

## Abstract

Unmodified single-stranded DNA has recently gained popularity for the templated synthesis of fluorescent noble metal nanoclusters (NCs). Bright, stable, and biocompatible clusters have been developed primarily through optimization of DNA sequence. However, DNA backbone modifications have not yet been investigated. In this work, phosphorothioate (PS) DNAs are evaluated in the synthesis of Au and Ag nanoclusters, and are employed to successfully template a novel emitter using T_15_ DNA at neutral pH. Mechanistic studies indicate a distinct UV-dependent formation mechanism that does not occur through the previously reported thymine N3. The positions of PS substitution have been optimized. This is the first reported use of a T_15_ template at physiological pH for AgNCs.

## 1. Introduction

The strong fluorescent intensity, relative stability, low toxicity and small size of noble metal nanoclusters make them an attractive replacement for organic fluorophores and semiconductor quantum dots in biological analysis, biological imaging and environmental monitoring [1,2,3,4,5,6]. The use of single-stranded DNA (ssDNA) as a synthetic template has gained popularity due to its commercial availability, biocompatibility, sequence dependent emission, and molecular recognition properties [2,4].

Despite the development of bright DNA-templated nanoclusters with emission wavelengths that range from the UV to NIR regions, our understanding of their structure, synthetic mechanism, and origin of fluorescence is currently limited. Past work has focused on the development and characterization of novel fluorescent clusters through the control of a variety of synthetic parameters, including nucleotide sequence, pH, redox conditions, and metal-DNA stoichiometry [3,4]. However, as of yet, DNA backbone modifications have not been examined.

**Figure 1:**
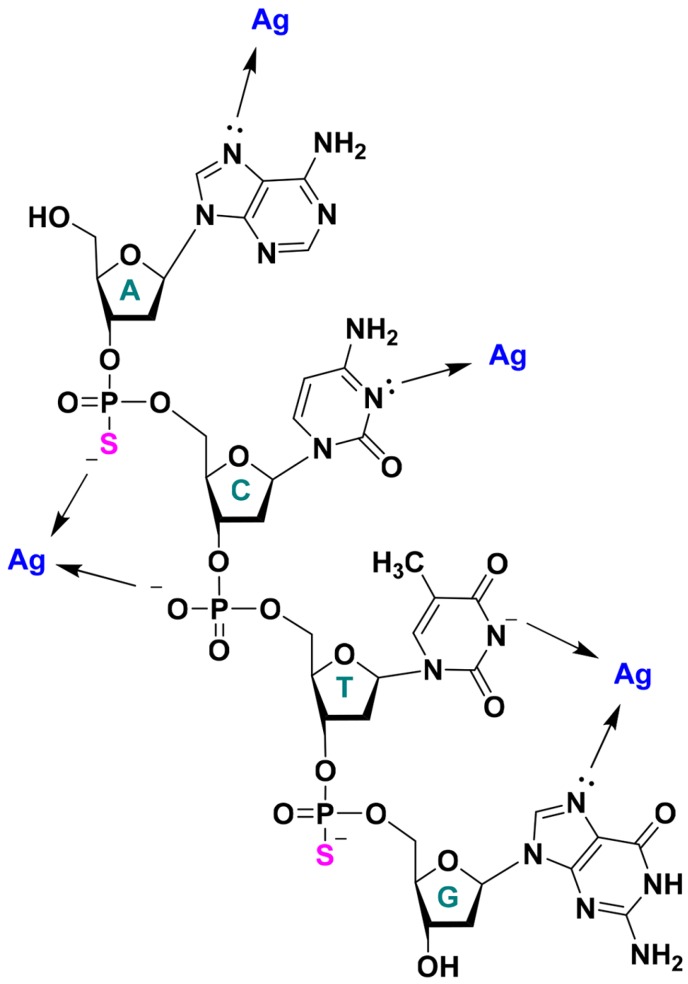
Schematic illustrating the potential sites of interaction between single stranded DNA and silver ions. Nucleobase interactions shown are based on previous literature reports. In this work, we explore the effect of backbone phosphorothioate modifications. Note that the thymine nitrogen needs to deprotonate for metal coordination.

Phosphorothioate DNA (PS DNA) is a DNA variant in which one of the non-bridging phosphate oxygen atoms is replaced by sulphur as shown in Figure 1. These modified oligonucleotides have become popular for designing antisense nucleic acids with high nuclease resistance and lipid bi-layer permeability, as well as for elucidating metal binding sites and ribozyme mechanisms [7,8,9]. More recently it has been employed in the assembly and positioning of metallic nanoparticles, and semi-conductor quantum dots, as well as in the development of chemical biology probes [10,11,12,13,14].

Previously, small thiol compounds such as glutathione have been shown to be effective in stabilizing fluorescent gold and silver nanoclusters [6,15]. There are also reports that thiol can both increase and decrease the emission intensity of nanoclusters templated by DNA [6,16]. Compared to a normal thiol modification, PS is much more cost-effective and can be introduced at multiple locations [7]. This substitution gives PS nucleic acids increased hydrophobicity, membrane permeability, oxidative stability, nuclease resistance and affinity for soft metal ions [7,17,18]. It has also been shown to induce conformational changes, which may be explained by the increased atomic radius of sulfur, and redistribution of electron density in the phosphodiester bond [19,20]. Despite these changes, PS modification leaves DNA charge density and nucleobase pK_a_ unchanged [18,21]. It is expected that PS DNA may confer novel optical properties and provide insight into the structure and properties of DNA-nanoclusters. The aim of this work will be to synthesize PS DNA stabilized fluorescent metal NCs, and investigate the effect of phosphorothioate modification of the DNA backbone on optimal synthetic conditions, as well as the physical, optical and chemical properties of templated metal NCs.

## 2. Results and Discussion

### 2.1. DNA Sequence Screening for AgNCs

The structure of a single-stranded DNA is shown in Figure 1. A noble metal ion or cluster may interact with the phosphate backbone via electrostatic interaction. Gold and silver typically interact with phosphate weakly. However, with a PS modification, an enhanced backbone interaction is expected. At the same time, they may also coordinate with DNA via the endocyclic nitrogens and exocyclic carbonyl-groups of the nucleobases as well as through interactions with the π-system of the aromatic rings [22]. While no advantageous properties were detected using PS-DNA in AuNC synthesis (see Table S1 and Figure S1), some benefits were seen with silver.

**Figure 2:**
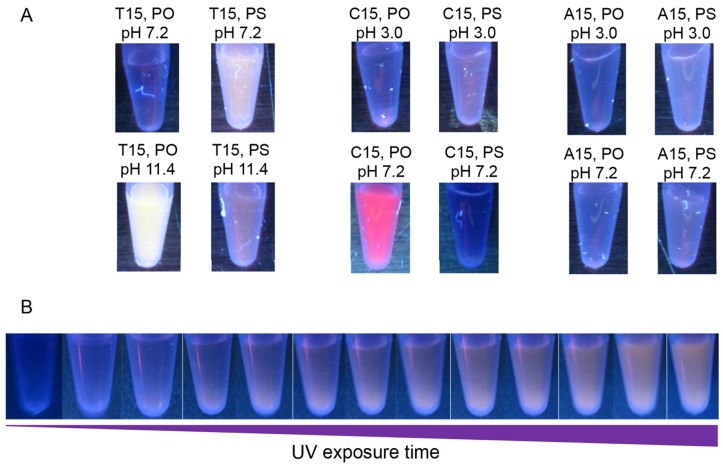
(**A**) Fluorescence photographs of phosphodiester (PO)- and phosphorothioate (PS)-modified 15-m DNA stabilized fluorescent AgNCs excited by a handheld UV lamp. Images were taken after exposure of samples to short wavelength UV light for approximately 5 min. Samples T_15_ pH 7.2 and 11.4, C_15_ pH 3, and A_15_ pH 3 and 7.2 were imaged under exposure to long wavelength UV light (365 nm). Samples of C_15_ pH 7.2 were imaged under short wavelength UV light (254 nm). Wavelength range of exposure did not impact the relative intensity of the two (PS and PO) samples; (**B**) Image sequence showing the increase in visible fluorescence of a T_15_-PS AgNC sample prepared at pH 7.2 under continuous exposure to short wavelength UV light. Exposure time from left to right is as follows (mm:ss): 00:00, 00:20, 00:46, 01:20, 01:44, 01:58, 02:12, 02: 24, 02:54, 03:18, 03:56, 05:38, 06:08. Sample was prepared using 15 μM DNA, 120 μM AgNO_3_, 120 μM NaBH_4_, and 25 mM pH 7.2 phosphate buffer.

In this synthesis, 15-m DNA was used for all AgNC synthesis. Base protonation has previously been shown to have a dramatic impact on template fluorescence [23,24]. Subsequently, C_15_, A_15_ and T_15_ homopolymers (see Table 1) were evaluated at a solution pH at least one unit greater than and less than the nucleobase pK_a_. The reduction was achieved with the standard NaBH_4_ at a ratio of NaBH_4_:Ag^+^ = 1:1, and the samples were observed under UV excitation (Figure 2A). Emission spectra were also recorded for each (see Figure S2). The behavior of the unmodified phosphodiester (PO)-DNA is consistent with the previous literature reports. For example, unmodified T_15_ produced strong fluorescence only at high pH, while unmodified C_15_ stabilized AgNCs emitted strongly at neutral pH. In these cases, PS modification negatively impacted the fluorescent properties of the clusters produced. However, improved fluorescence was observed for PS templates under two conditions. A_15_-PS was able to produced weak, but slightly improved visual emission at both low and neutral pH (Figure 2, Figure S2C,D) and more impressively, PS modification enabled the formation of a previously unreported T_15_-templated violet emitter at neutral pH, resulting in increased overall visual fluorescence. PS-modification of C_15_ at pH 3 resulted in a slight shift in emission wavelength, but no enhancement in fluorescence (Figure S2A).

**Table 1:** DNA sequences used as templates in nanocluster synthesis (from 5'-terminus).

DNA Name	Sequence and Modifications
A_15_-PO	AAAAAAAAAAAAAAA
A_15_-PS	A*A*A*A*A*A*A*A*A*A*A*A*A*A*A
C_15_-PO	CCCCCCCCCCCCCCC
C_15_-PS	C*C*C*C*C*C*C*C*C*C*C*C*C*C*C
T_15_-PO	TTTTTTTTTTTTTTT
T_15_-PS	T*T*T*T*T*T*T*T*T*T*T*T*T*T*T
T_15_-PS1	TTTTTTTT*T*T*T*T*T*T*T
T_15_-PS2	T*TT*TT*TT*TT*TT*TT*TT

* = PS modification.

PS modification may have been detrimental to previously templated strong emitters for several reasons. PS modification may either stabilize the formation of distinct, non-fluorescent species, or it may simply be quenching the fluorescence of the clusters produced with unmodified templates. Thiol compounds have previously been found to quench the fluorescence of some DNA-AgNCs via electron donation from the sulfur lone-pair to a low lying d-orbital on silver [25]. In one case, using C_12_ DNA fluorescence was actually enhanced by biothiols, and changes circular dichromism spectra indicated a formation of a more compact DNA structure in addition to formation of S-Ag bonds [26]. However, the present data indicates that this effect does not occur with PS modification.

Since our aim is to find sequences where the PS DNA produced stronger fluorescence than the corresponding PO DNA. T_15_ DNA was chosen to be studied in more detail.

### 2.2. Different PS Compositions

For the above work, PS modification was introduced at each phosphate position. To further optimize DNA PS composition, two partially phosphorothioated T_15_ templates were evaluated to determine the effect of number and position of PS modifications on cluster fluorescence. One template (T_15_-PS1, Table 1) possessed seven sequential PS linkages at the 3'-end of the template, while the other (T_15_-PS2) had the same number of PS modifications but on every other linkage such that they were uniformly dispersed along the T_15_ strand. Samples templated with T_15_-PO, T_15_-PS1, T_15_-PS2, and T_15_-PS were prepared using 15 µM DNA, 120 µM AgNO_3_ and 120 µM NaBH_4_ in 25 mM phosphate buffer at both pH 7.2 and pH 11.4. The samples were incubated in dark for 2 h after adding NaBH_4_ and then exposed to UV light for 10 min before measuring the spectra.

**Figure 3:**
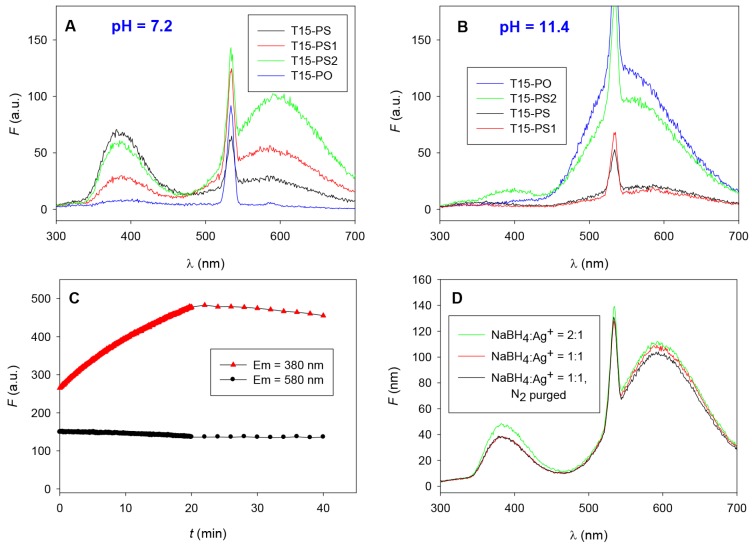
Emission spectra (λ_EX_ 266 nm) of fully-, partially-, and non-phosphorothioate modified T_15_ DNA template at (**A**) pH 7.2 and (**B**) pH 11.4; Samples were prepared using 15 µM DNA, 120 µM AgNO_3_ and 120 µM NaBH_4_ in 25 mM phosphate buffer at pH 7.2 or 11.4 and incubated in the dark for 2 hours. Spectra were measured following exposure under a handheld UV lamp at short wavelength for 10 min. Note that an excitation wavelength of 266 nm was chosen for its ability to produce emission from both bands, and favors emission from the yellow-orange band; (**C**) Emission intensities of T_15_-PS-templated sample at 380 nm and 580 nm of samples under continuous excitation in fluorescence spectrophotometer at 266 nm using a 20 nm excitation slit and 1 s averaging time, followed by storage in the dark with a 1 s measurement taken every 2 min. UV exposure stopped after 20 min. The sample was prepared using 30 μM DNA, 240 μM AgNO_3_ and 240 μM NaBH_4_; (**D**) T_15_-PS2, pH 7.2 AgNC samples prepared using different NaBH_4_ concentrations and the effect of N_2_ purging to remove oxygen. The sharp peak at 532 nm is due to the low efficiency of monochromators to block light at half frequency of the excitation wavelength.

Across the four templates and two pH values, two major fluorescent bands were observed with varying intensity (Figure 3A,B). A violet emitter, λ_EM_ Max ~ 380 nm, λ_EX_ Max ~ 325 nm, was favored by neutral pH (7.2) and PS-modified templates, and a yellow-orange emitter λ_EM_ Max ~ 580 nm, λ_EX_ Max ~ 266 nm was favored by high pH (11.4) and unmodified PO-templates. Since the N3 position of thymine is deprotonated across this range (pK_a_ ~ 9.9), binding through this position may be involved in the formation of the yellow-orange emitter, but not the violet emitter. This may indicate that the violet emitter is produced through a pH insensitive interaction with the PS backbone, but is overcome by formation of the more favorable yellow-orange emitter when enabled via deprotonation at the N3 position.

One feature of note is that regardless of the degree or pattern of PS modification, only two distinct emitters are formed. This suggests that PS modification alters the favorability of forming a distinct species, rather than simply altering the template geometry or electronic environment surrounding the cluster which would be expected to cause a more gradual change in fluorescent properties. T_15_-PS2 with alternating PS modifications was able to template both PO- and PS-favored species and produced the greatest overall fluorescence. As a result, it was used to investigate the conditions leading to the formation of the two fluorescent species.

### 2.3. UV-Light Activated Fluorescence

It needs to be noted that all the above T_15_-PS DNA data shown above were collected after exposing the samples to UV light. When we observed under excitation by short wavelength handheld UV light, T_15_-PS-DNA-templated samples, were initially non-fluorescent, but evolved increasing fluorescence upon continuous exposure over a period of approximately 5 min as seen in Figure 2B. To quantitatively evaluate the effect of light exposure, fluorescent intensity (λ_EM_ 380 nm and 580 nm) of each sample was measured under continuous excitation at 266 nm in the fluorescence spectrophotometer using a 20 nm slit width and 1 s averaging time (Figure 3C). The photo-induced emission was the most pronounced for the violet, 380 nm peak, templated by T_15_-PS DNA at neutral pH. The photo induced emission effect was also evaluated for the T_15_-PO template, but little increase was observed at high pH, and no fluorescence at all was observed at neutral pH (data not shown). This suggests that the effect is strongly favored by PS DNA, potentially due to the additional binding geometries or aggregation states enabled by this modification. After 20 min, the UV exposure was stopped and the peak also stopped increasing.

The observation that both emission bands either increase or remain constant under UV exposure suggests that the photo-induced emission does not result from a direct photo-oxidative conversion. This inter-conversion was further investigated later, by preparing samples using two times the concentration of NaBH_4_ as well as using a nitrogen purged solution. Oxygen has previously been shown essential in the formation of certain partially oxidized species [24]. However, in this case neither condition has any significant effect on the relative distribution of the two species (Figure 3D), suggesting both species might be fully reduced.

### 2.4. Effect of Heat and Light on Kinetics of Cluster Formation

This is the first reported incidence of photo-induced fluorescence in a DNA-AgNC. Light has however, been found essential in the formation of luminescent gold particles as formed in the presence of adenine derivatives [27]. It was suggested in that study suggested that light may be necessary to induce a re-organization of gold atoms into a productive geometry, and it was found that heating could achieve the same goal. In order to investigate the mechanism of producing the two emission bands and to identify reproducible synthetic conditions, a few experiments were carried out. A T_15_-PS2 template, possessing alternating PS linkages, and a pH of 7.2 was chosen for reaction conditions due to their ability to produce both emission bands with sufficient intensity. The evolution of fluorescence was measured as a function of incubation time under exposure to the following conditions: short wavelength UV light, ambient fluorescent light, dark at room temperature, and dark at 75 °C. Comparison of the conditions producing the two bands suggests unique formation mechanisms for each.

**Figure 4:**
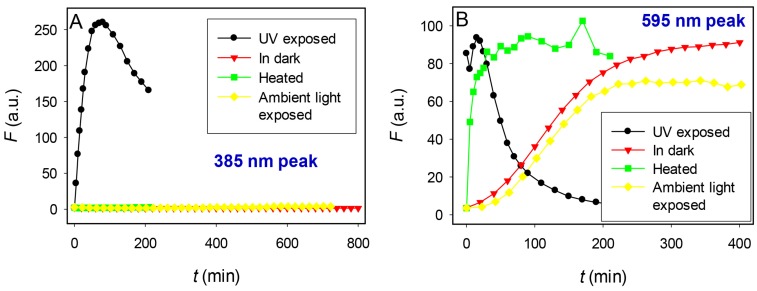
Evolution of T_15_-PS2-AgNC fluorescence from the (**A**) violet band (λ_EM_ 385 nm) and (**B**) yellow-orange band (λ_EM_ 595 nm) as a function of incubation under UV exposure (**black**), in the dark at room temperature (**red**), while heated at 75 °C in the dark (**green**), and under exposure to ambient fluorescent light (**yellow**). Samples were prepared using 15 μM DNA, 120 μM AgNO_3_ and 120 μM NaBH_4_.

For the yellow-orange emitter (λ_EM_ 595 nm) UV light (black, Figure 4B) appeared to provide activation energy in the formation of the fluorescent species, rapidly increasing the reaction rate compared to that in the dark (red, Figure 4B), but having no effect on absolute intensity. This activation energy could also be supplied by heating at 75 °C (green, Figure 4B), but not by ambient fluorescent light (yellow, Figure 4B) which slightly reduced the emission intensity (21%) and had no appreciable effect on the reaction rate.

In the case of the violet emitter (λ_EM_ 385 nm), UV light was essential (black, Figure 4A), and essentially no fluorescence was observed under ambient light, or in the dark, with or without heat (yellow, green and red, Figure 4A). This suggests that UV irradiation is responsible for this short wavelength emission and the sulfur atom is critical for this activation. The exact mechanism of action remains to be explored. Under UV light, the violet band (black, Figure 4A) took longer (~40 min) to reach maximum intensity, compared to the yellow-orange band (black, Figure 4B) which reached an intensity maximum after only 5 min, but also exhibited greater photostability, maintaining 50% of its emission intensity approximately 230 min after reaching its peak compared to only approximately 30 min for the yellow-orange band. Although UV exposure was essential, it was not sufficient, and in the absence of NaBH_4_, only very faint violet emission was observed, even after 5 h exposure to short wavelength short wavelength UV light (data not shown).

## 3. Experimental Section

### 3.1. Reagents

Oligonucleotides were purchased from Eurofins MWG Operon (Huntsville, AL, USA), or Integrated DNA Technologies (Coralville, IA, USA) and were purified via standard desalting by the manufacturer.

Citric acid monohydrate (Sigma Aldrich, St. Louis, MO, USA, 99.0%), citric acid trisodium dihydrate (Amresco, Solon, OH, USA, reagent grade), HEPES (Amresco, Solon, OH, USA, high purity grade), sodium phosphate monobasic dihydrate (Fisher, Waltham, MA, USA, 100.0%), sodium phosphate dibasic heptahydrate (Fisher, Waltham, MA, USA, 99.0%–102.0%), sodium hydroxide (Amresco, Solon, OH, USA, ACS grade), nitric acid (Fisher, Waltham, MA, USA, ACS reagent grade), tetrachloroauric acid trihydrate (Fisher, Waltham, MA, USA, ACS Reagent Grade, ≥49% Au Basis, equivalent of 98% HAuCl_4_), silver nitrate (Alfa Aesar, Ward Hill, MA, USA, 99.9%) and sodium borohydride (EMD, Billerica, MA, USA, 98.0%) were used as is. All solutions were prepared using ultrapure water (18.2 MΩ·cm at 25 °C, Synergy UV water purification system, Millipore Co., Billerica, MA, USA).

### 3.2. AgNC Synthesis

Buffer, DNA and AgNO_3_ were dissolved in ultrapure water and vortexed. After approximately 5 min, freshly prepared NaBH_4_ was added. The reaction mixture was then again vortexed, and incubated for approximately 2 h in the dark at room temperature. Typical reagent concentrations for AgNC synthesis were as follows: 25 mM buffer, 15 µM DNA, 120 µM AgNO_3_, and 120 µM NaBH_4_. Where indicated, samples were then exposed to short wavelength UV light prior to characterization.

### 3.3. Fluorescence Spectroscopy

Visible Fluorescence. Fluorescence was assessed visually under excitation under a hand held UV lamp at 254 nm or 365 nm in a dark room and recorded using a digital camera (Canon PowerShot SD1200 IS). Samples were also observed under ambient light. Full excitation and emission spectra were typically measured without dilution using a Varian Eclipse Fluorescence Spectrophotometer (Agilent Technologies, Santa Clara, CA, USA). Emission spectra were first measured using an excitation wavelength of 300 nm. Excitation spectra were then measured for emission maxima observed, and finally, emission spectra were again recorded for the measured excitation maxima.

## 4. Conclusions

In this work, we have investigated the effect of PS-modified ssDNA templates in the synthesis of fluorescent gold and silver nanoclusters. While PS modification did not bring stronger emission in the synthesis of AuNCs, PS-modified T_15_ template enabled the formation of stronger violet and yellow-orange emitters at neutral pH whereas unmodified T_15_ had previously only produced fluorescence at high pH. The insensitivity of this emitter to base protonation and the favorability of PS modifications suggests that DNA-Ag interaction may occur through the PS backbone. However, the synthesis is not completely independent of base sequence, as strong fluorescence could not be achieved using any other templates. The present work is the first time that a poly-T template has been used successfully to template fluorescent species at neutral pH, potentially extending the use of T-rich templates for physiological conditions.

## Supplementary Materials

Supplementary materials can be accessed at: http://www.mdpi.com/2079-4991/5/2/804/s1.

## References

[1] Díez I., Ras R.H.A. (2011). Fluorescent silver nanoclusters. Nanoscale.

[2] Liu J. (2014). DNA-stabilized, fluorescent, metal nanoclusters for biosensor development. Trends Anal. Chem..

[3] Shang L., Dong S., Nienhaus G.U. (2011). Ultra-small fluorescent metal nanoclusters: Synthesis and biological applications. Nano Today.

[4] Zhang L., Wang E. (2014). Metal nanoclusters: New fluorescent probes for sensors and bioimaging. Nano Today.

[5] Wei H., Wang Z., Yang L., Tian S., Hou C., Lu Y. (2010). Lysozyme-stabilized gold fluorescent cluster: Synthesis and application as Hg^2+^ sensor. Analyst.

[6] Yuan Z., Peng M., Shi L., Du Y., Cai N., He Y., Chang H.T., Yeung E.S. (2013). Disassembly mediated fluorescence recovery of gold nanodots for selective sulfide sensing. Nanoscale.

[7] Deleavey G.F., Damha M.J. (2012). Designing chemically modified oligonucleotides for targeted gene silencing. Chem. Biol..

[8] Sekhon G.S., Sen D. (2010). A stereochemical glimpse of the active site of the 8–17 deoxyribozyme from iodine-mediated cross-links formed with the substrate’s scissile site. Biochemistry.

[9] Wang S., Karbstein K., Peracchi A., Beigelman L., Herschlag D. (1999). Identification of the hammerhead ribozyme metal ion binding site responsible for rescue of the deleterious effect of a cleavage site phosphorothioate. Biochemistry.

[10] Tan L.H., Xing H., Lu Y. (2014). DNA as a powerful tool for morphology control, spatial positioning, and dynamic assembly of nanoparticles. Acc. Chem. Res..

[11] Ma N., Sargent E.H., Kelley S.O. (2009). One-step DNA-programmed growth of luminescent and biofunctionalized nanocrystals. Nat. Nanotechnol..

[12] Huang P.J.J., Liu J. (2014). Sensing parts-per-trillion Cd^2+^, Hg^2+^, and Pb^2+^ collectively and individually using phosphorothioate DNAzymes. Anal. Chem..

[13] Zhang D., Deng M., Xu L., Zhou Y., Yuwen J., Zhou X. (2009). The sensitive and selective optical detection of mercury(II) ions by using a phosphorothioate DNAzyme strategy. Chem. Eur. J..

[14] Lee J.H., Wernette D.P., Yigit M.V., Liu J., Wang Z., Lu Y. (2007). Site-specific control of distances between gold nanoparticles using phosphorothioate anchors on DNA and a short bifunctional molecular fastener. Angew. Chem. Int. Ed. Engl..

[15] Link S., Beeby A., FitzGerald S., el-Sayed M.A., Schaaff T.G., Whetten R.L. (2002). Visible to infrared luminescence from a 28-atom gold cluster. J. Phys. Chem. B.

[16] Han B., Wang E. (2012). DNA-templated fluorescent silver nanoclusters. Anal. Bioanal. Chem..

[17] Xie X., Liang J., Pu T., Xu F., Yao F., Yang Y., Zhao Y.L., You D., Zhou X., Deng Z. (2012). Phosphorothioate DNA as an antioxidant in bacteria. Nucleic Acids Res..

[18] Guga P., Koziołkiewicz M. (2011). Phosphorothioate nucleotides and oligonucleotides—Recent progress in synthesis and application. Chem. Biodivers..

[19] Cruse W.B., Salisbury S.A., Brown T., Cosstick R., Eckstein F., Kennard O. (1986). Chiral phosphorothioate analogues of B-DNA. The crystal structure of Rp-d[Gp(S)CpGp(S)CpGp(S)C]. J. Mol. Biol..

[20] Smith J.S., Nikonowicz E.P. (2000). Phosphorothioate substitution can substantially alter RNA conformation. Biochemistry.

[21] Moody E.M., Brown T.S., Bevilacqua P.C. (2004). Simple method for determining nucleobase pK_a_ values by indirect labeling and demonstration of a pK_a_ of neutrality in dsDNA. J. Am. Chem. Soc..

[22] Liu J. (2012). Adsorption of DNA onto gold nanoparticles and graphene oxide: Surface science and applications. Phys. Chem. Chem. Phys..

[23] Ritchie C.M., Johnsen K.R., Kiser J.R., Antoku Y., Dickson R.M., Petty J.T. (2007). Ag nanocluster formation using a cytosine oligonucleotide template. J. Phys. Chem. C.

[24] Sengupta B., Ritchie C.M., Buckman J.G., Johnsen K.R., Goodwin P.M., Petty J.T. (2008). Base-directed formation of fluorescent silver clusters. J. Phys. Chem. C.

[25] Han B., Wang E. (2011). Oligonucleotide-stabilized fluorescent silver nanoclusters for sensitive detection of biothiols in biological fluids. Biosens. Bioelectron..

[26] Huang Z., Pu F., Lin Y., Ren J., Qu X. (2011). Modulating DNA-templated silver nanoclusters for fluorescence turn-on detection of thiol compounds. Chem. Commun..

[27] Lopez A., Liu J. (2013). Light-activated metal-coordinated supramolecular complexes with charge-directed self-assembly. J. Phys. Chem. C.

