# Supplementary material for: Phosphorothioate DNA Stabilized Fluorescent Gold and Silver Nanoclusters
Source: Nanomaterials (Basel). 2015 May 19;5(2):804–13. doi: 10.3390/nano5020804 (PMC5312886; doi:10.3390/nano5020804)
Supplement: Supplementary file 2 [file nanomaterials-05-00804-s002.pdf]

Supplementary Information

S.1. PS DNA Stabilized AuNCs

Small thiolated compounds have a strong affinity for both neutral and ionic gold atoms, and have been widely used as reducing agents and stabilizers in the synthesis of AuNCs [1]. In comparison to DNA however, thiol stabilizers lack the 3D structure that allows greater control over size distribution and the ability to modulate emission through variations in base sequence. Here, we expect that PS modifications may provide new coordination chemistry in DNA for preparing new fluorescent AuNCs. Our previous work indicates that shorter DNA produces stronger fluorescence [2]. To investigate the utility of PS DNA in AuNC synthesis, A_5, T_5, C_5 and G_5 templates (Table S1), both without and with full PS modification were screened using conditions based on those previously employed.

Table S1. DNA sequences used as templates in AuNC synthesis (from 5'-terminus).

DNA Name	Sequence and Modifications
A_5-PO	AAAAA
A_5-PS	A*A*A*A*A
T_5-PO	TTTTT
T_5-PS	T*T*T*T*T
C_5-PO	CCCCC
C_5-PS	C*C*C*C*C
G_5-PO	GGGGG
G_5-PS	G*G*G*G*G

* = PS modification

In a typical synthesis, 65 μM of DNA (see Table 1) was dissolved in ultrapure water. $HAuCl_4$ was added to give a Au^{3+}:base ratio of 1:1.3, and the mixture was vortexed. Then, citrate buffer was added to give a final concentration of approximately 50 mM, the sample was again vortexed, and incubated in the dark for a minimum of 8 hours or overnight.

Following our previous work [2,3], fluorescent AuNCs were produced using both A_5-PO and A_5-PS templates using a pH 6 citrate solution as a buffer and reducing agent. A_5-PO and A_5-PS produced emission bands at 465 nm and 429 nm respectively as seen in Figure S1A. Although successful, the overall emission intensity of the A_5-PS template clusters was approximately half that of its PO counterpart. PS modification of the A_5 template also induced a red-shift in excitation wavelength (300 nm → 353 nm) and a blue-shift in emission wavelength (465 nm → 429 nm), resulting in a significantly reduced Stokes shift.

Next, we tested C_5 DNA using citrate as both buffer and reducing agent. Previous work indicates that lower pH works best for poly-C DNA templated AuNCs, and here we also found that fluorescence was produced with the PO-DNA at lower pH (Figure S1B). Although a small hump was observed for the C_5-PS template, it is significantly weaker than the C_5-PO, pH 3 emission band. At neutral pH, the emission was poor for both PO and PS C_5 DNA (data not shown).

When T_5 and G_5 were tested, no strong emission was observed for either sample (data not shown). Therefore, overall, PS modification did not enhance the emission property of DNA templated AuNCs, although fluorescent AuNCs can still be produced with unmodified poly-A and poly-C DNA.

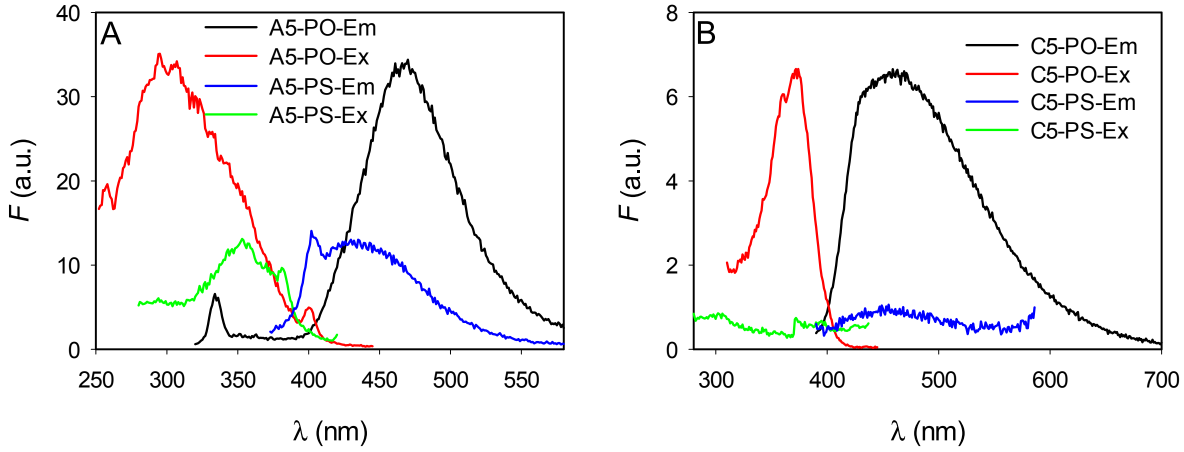

Figure S1. Fluorescence excitation and emission spectra of the (**A**) A_5 DNA at pH 6 (PO and PS) and (**B**) C_5 DNA at pH 3 (PO and PS) stabilized fluorescent AuNCs. The excitation and emission wavelengths are at the corresponding peak positions. Spectra were acquired after 8 hours dark incubation, on 1:2.5 dilutions in ultrapure water. The synthesis was carried out in citrate buffer with 65 μM DNA and 250 μM $HAuCl_4$. The small peak at approximately 400 nm (Figure S1A) can be attributed to water Raman scattering.

S.2. PS DNA Stabilized AgNCs

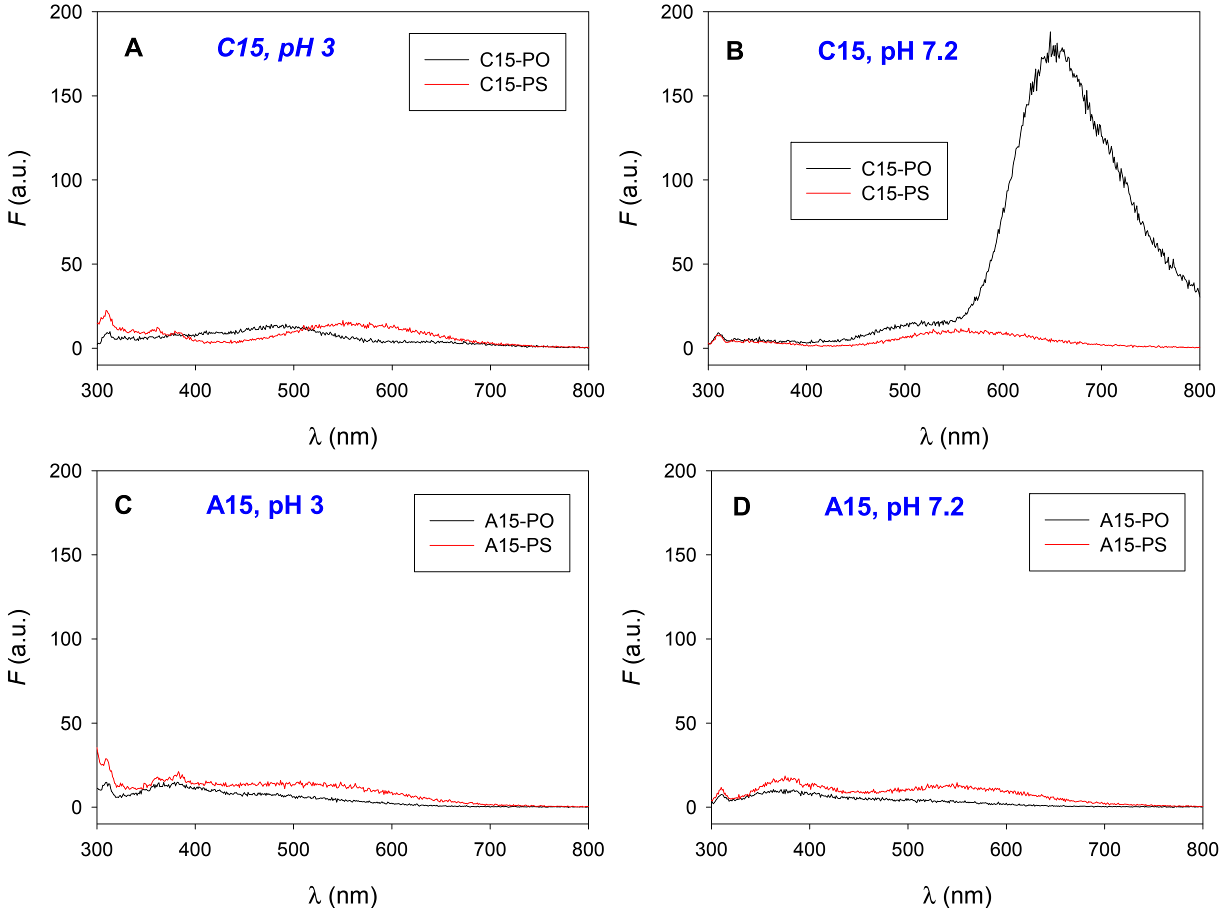

Figure S2. *Cont.*

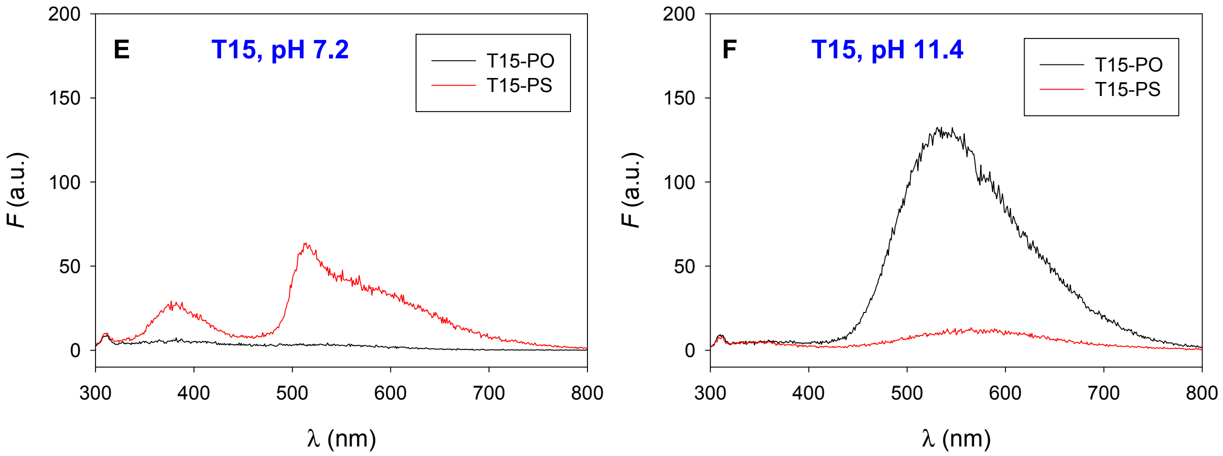

Figure S2. Emission spectra (λ_{EX} 280 nm) of PO- and PS-modified (**A**) C_{15} at pH 3; (**B**) C_{15} at pH 7.2; (**C**) A_{15} at pH 3; (**D**) A_{15} at pH 7.2; (**E**) T_{15} at pH 7.2; and (**F**) T_{15} at pH 11.4. Samples were prepared using 15 µM DNA, 120 µM $AgNO_3$, 120 µM $NaBH_4$, and 25 mM pH 7.2 phosphate buffer. Spectra were recorded on undiluted samples following 2 h dark incubation followed by exposure to hand held short wavelength UV light for 5 min.

References

1. Cui, M.; Zhao, Y.; Song, Q. Synthesis, optical properties and applications of ultra-small luminescent gold nanoclusters. *Trends Anal. Chem.* **2014**, *57*, 73–82.
2. Lopez, A.; Liu, J. DNA templated fluorescent gold nanoclusters reduced by Good's buffer: From blue emitting seeds to red and near infrared emitters. *Can. J. Chem.* **2015**, doi:10.1139/cjc-2014-0600.
3. Kennedy, T.A.C.; MacLean, J.L.; Liu, J. Blue emitting gold nanoclusters templated by poly-cytosine DNA at low pH and poly-adenine DNA at neutral pH. *Chem. Commun.* **2012**, *48*, 6845–6847.

© 2015 by the authors; licensee MDPI, Basel, Switzerland. This article is an open access article distributed under the terms and conditions of the Creative Commons Attribution license (http://creativecommons.org/licenses/by/4.0/).
